# Effect and mechanism of eugenol on storage quality of fresh-peeled Chinese water chestnuts

**DOI:** 10.3389/fpls.2022.965723

**Published:** 2022-09-28

**Authors:** Zhe Chen, Yuhan Xu, Yang Lu, Zeyu Miao, Yang Yi, Limei Wang, Wenfu Hou, Youwei Ai, Hongxun Wang, Ting Min

**Affiliations:** ^1^ College of Food Science and Engineering, Wuhan Polytechnic University, Wuhan, China; ^2^ College of Biology and Pharmaceutical Engineering, Wuhan Polytechnic University, Wuhan, China

**Keywords:** eugenol, reactive oxygen metabolism, phenolic metabolism, storage quality, fresh-peeled Chinese water chestnut

## Abstract

The study aimed to investigate the effect and mechanism of eugenol treatment on fresh-peeled Chinese water chestnuts (CWCs). The results found that eugenol treatment maintained the appearance of fresh-peeled CWCs, accompanied by higher L* value, total solids and O_2_ contents, as well as lower browning degree, weight loss rate, CO_2_ content, a* and b* values. In addition, eugenol treatment significantly reduced the activities of peroxidase, phenylalanine ammonia-lyase, and polyphenol oxidase, as well as the total content of soluble quinone in fresh-peeled CWCs. Meanwhile, fresh-peeled CWCs treated with eugenol showed markedly lower content of total flavonoids, which may be related to yellowing. Furthermore, eugenol treatment suppressed the rates of O_2_·^-^ and OH·^-^ production as well as the contents of H_2_O_2_ and malondialdehyde in fresh-peeled CWCs. During the storage, eugenol treatment not only increased the activities of catalase, superoxide dismutase, ascorbate peroxidase and glutathione reductase as well as the DPPH free radical scavenging rate, but also increased the total phenolics, ascorbic acid and glutathione contents. In summary, eugenol treatment delayed the surface discoloration of fresh-peeled CWCs by improving the antioxidant capacity, inhibiting the phenolic compound metabolism and scavenging ROS, thus effectively maintaining the quality of fresh-peeled CWCs while extending their shelf life.

## Introduction

Chinese water chestnuts (CWCs) grow mainly in the south area of the Yangtze River in China (Nie et al., 2021), and are rich in starch, vitamins, minerals, and proteins (Jiang et al., 2004). CWCs are usually peeled before being eaten, due to a certain quantity of pathogenic microorganisms are attached to their peels (Jiang et al., 2004). As a result, fresh-peeled CWCs are increasingly welcomed among consumers for deliciousness and convenience. However, CWCs are often vulnerable to mechanical damage in the fresh cutting process, resulting in the decline of quality and reduction of the shelf life. Surface discoloration is an important phenomenon indicating a quality decrease in fresh-peeled CWCs (Song et al., 2019).

There are hypotheses on the discoloration of fresh-peeled CWCs. One hypothesis is that the surface discoloration is yellowing. Flavonoids, especially naringin and eriodictyol, are the main yellowing substances in fresh-peeled CWCs (Pan et al., 2015). The other hypothesis is that enzymatic browning contributes to surface discoloration, and phenylalanine ammonia-lyase (PAL), peroxidase (POD), and polyphenol oxidase (PPO) are key enzymes (Li et al., 2020). The senescence of fresh-cut vegetables and fruits has been reported to be closely related to ROS, like H_2_O_2_, O_2_·^-^ and OH·^-^. Excessive accumulation of ROS disrupts the balance of the scavenging system, which gives rise to malondialdehyde (MDA) accumulation and membrane lipid peroxidation, further exacerbating the senescence process (Dou et al., 2021). Moreover, ROS metabolism is also closely related to the activities of antioxidant enzymes (Li et al., 2021). The antioxidant system is mainly composed of ascorbic acid (AsA), glutathione (GSH), catalase (CAT), superoxide dismutase (SOD), ascorbate peroxidase (APX) and glutathione reductase (GR) and other enzymes. The main function of CAT is to generate H_2_O and O_2_ by specifically catalyzing the decomposition of H_2_O_2_. The catalytic effect of SOD is to inhibit the damage of O_2_·^-^ to the plant, establishing the first defensive line of the antioxidant system. The important antioxidant enzymes in the ascorbic acid-glutathione cycle are APX and GR, which work together with antioxidants to remove ROS and protect the integrity of cell membranes. And it is reported that lower accumulation of ROS and higher antioxidative enzyme activity play a role in alleviating the discoloration of fresh-peeled CWCs treated with hydrogen sulfide (Dou et al., 2021).

Therefore, it is a matter of great concern to seek effective methods to delay the discoloration and quality deterioration of fresh-peeled CWCs. Various methods have been currently adopted to maintain the appearance of fresh-peeled CWCs and to prolong the shelf life. Anoxic treatment contributes to alleviating lipid peroxidation and maintaining storage quality in fresh-peeled CWCs by reducing the activity of malondialdehyde, H_2_O_2_ and lipoxygenase and increasing the activity of ascorbate peroxidase and superoxide dismutase (You et al., 2012). Pen and colleagues used chitosan coating treatment to delay discoloration of fresh-peeled CWCs, reducing the activity of PAL, PPO, and POD as well as the content of total phenolics (Jiang et al., 2004). Hydrogen-rich water treatment delayed the yellowing of fresh-peeled CWCs, alleviated oxidative damage, and decreased the PAL activity and accumulation of flavonoids (Li et al., 2022). Moreover, ferulic acid is effective in inhibiting yellowing and eriodictyol and naringenin levels in fresh-peeled CWCs (Song et al., 2019). Salicylic acid also inhibits the browning of fresh-peeled CWCs and reduces the activity of PPO, POD, and PAL (Peng and Jiang, 2006). At present, the main treatment method is concentrated on chemical preservatives. Today, with the increasing requirements for food safety, the development of pure natural plant extracts from plants to extract antibacterial and bactericidal active substances has been put on the agenda (Fernández-Pan et al., 2012; Alshaikh and Perveen, 2017).

As a natural plant extract, eugenol is the main component of clove oil and has strong insecticidal, antibacterial, and antiseptic effects (Devi et al., 2013; Ma et al., 2016). EUG is a food additive approved by the U.S. FDA (Food and Drug Administration), with the LD_50_ value being 3000 mg kg ^-1^ oral mice (Gong et al., 2016). Eugenol is superior to hydrogen sulfide in safety and antimicrobial activity. And the production cost of eugenol is lower than those of ferulic acid and hydrogen-rich water. In the food industry, eugenol is mainly used for retaining the texture, sensory properties, and moisture of shrimp (Sharifimehr et al., 2019) and pork (Wan et al., 2018). However, there is little research on the storage and preservation of fresh-cut vegetables and fruits treated with eugenol. Fresh-cut lettuce treated with eugenol at the concentration of 0.5 g/L inhibited the browning of fresh-cut lettuce and the activity of PAL, PPO, and POD enzymes (Chen et al., 2017). Edible coatings with 1 g/L eugenol and 1.5 g/L citral on raspberries (Guerreiro et al., 2016) and fresh-cut apples (Guerreiro et al., 2016) have high Trolox equivalent antioxidant activity. Moreover, 15 g/L eugenol emulsion treatment delayed the browning of fresh-cut CWCs, enhanced the activities of ROS scavenging enzymes, and increased the contents of phenolic substances, thereby reducing the damage to the cell membrane (Teng et al., 2020; Zhu et al., 2022). Previous studies have shown that antioxidant and ROS metabolism are involved in the browning and senescence of fresh-cut CWCs (Dou et al., 2021). In addition to the direct antioxidant system, the ascorbic acid-glutathione (AsA-GSH) cycle plays an important role in maintaining the quality of fresh-cut fruits and vegetables. Therefore, the mechanism of eugenol on inhibiting discoloration and quality deterioration of fresh-peeled CWCs needs to be further investigated, especially the combined study of direct and indirect antioxidant processes.

This study investigated the effect of 1 g/L eugenol treatment on the quality of fresh-peeled CWCs during storage, and its underlying mechanism on phenolics and ROS metabolism. The quality change (color difference, browning degree, total soluble solids, weight loss rate, and headspace gas composition) of packaged fresh-peeled CWCs were evaluated first. To further understand the possible mechanism for discoloration of fresh-peeled CWCs, the phenolic metabolism (total flavonoid, total phenolic and total quinone contents, and activities of POD, PPO, and PAL), ROS metabolism (O_2_·^-^ and OH·^-^production rates, H_2_O_2_ and MDA contents), and antioxidant substances (activities of CAT, SOD APX, GR and DPPH free radical scavenging rate) were studied. This study aims to provide a theoretical basis for storing and preserving fresh-peeled CWCs with eugenol.

## Materials and methods

### Materials and reagents

**Figure 1:**
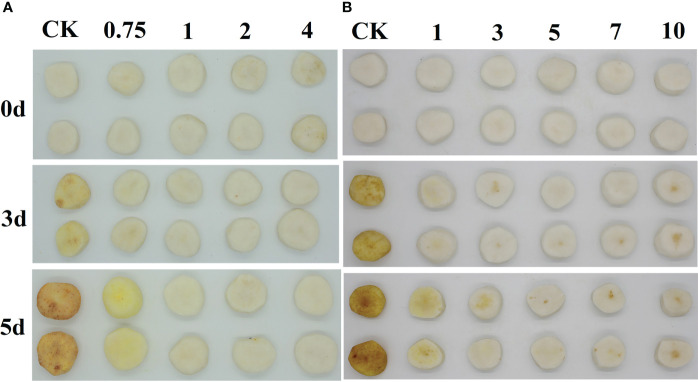
Images of 0, 0.75, 1, 2, and 4 g/L EUG treatment on fresh-peeled CWC **(A)**, and 0, 1, 3, 5, 7 and 10 min EUG treatment on fresh-peeled CWC **(B)**.

CWCs were bought from the Southeast Fruit Wholesale Market in Wuhan, Hubei. Severely damaged CWCs were removed and fruits with uniform size and appearance were selected as experimental materials. After pre-cooling at 4°C for 24 h, the CWCs were manually washed and peeled. Later, fresh-peeled CWCs were soaked in 0.1 g/L sodium hypochlorite for 5 min. The four eugenol concentrations were selected to be 0.75, 1, 2, and 4 g/L, and the five eugenol treatment times were 1, 3, 5, 7, and 10 min, respectively. It can be seen from **Figure 1A and B** that the optimal concentration of eugenol treatment is 1 g/L and the optimal time was 5 min. Therefore, one group was soaked in 1 g/L eugenol solution (Yuanye, Wuhan Feiyang Biological Technology Co., Ltd.) for 5 min, one group as the control was soaked in distilled water for 5 min, and the other group as the ethanol control was soaked in 5% ethanol for 5 min. Eugenol solution (1 g/L) was dissolved in a small amount of ethanol (5%) and diluted with distilled water. After draining the water, three groups of fresh-peeled CWCs were sealed in polyethylene bags (200 × 280 mm) after being placed in food-grade polyethylene trays (180 × 120 × 25 mm). At last, samples were stored at 10°C with a relative humidity of 85-90% (Fan et al., 2018) for 5 d and used for analysis at 1 d interval.

### Color difference, browning degree, total soluble solid content, weight loss rate, and headspace gas composition

An EOS550D camera was used to record the appearance of fresh-peeled CWCs. According to Du et al. (2009), a JZ-300 colorimeter was used to measure color differences (L*, a*, and b* values) of fresh-peeled CWCs.

The degree of browning fresh-peeled CWCs was measured using the method of Min et al. (2017). Three grams of fresh-peeled CWCs pulp was homogenized in 30 mL of distilled water, followed by centrifugation of the mixture at 10,000 ×g for 10 min. Later, the supernatant (3.5 mL) was incubated at 25°C for 5 min and the absorbance was determined at 410 nm by an A360 UV-Vis spectrophotometer (Aoyi Instrument Shanghai Co., Ltd).

The total soluble solid content of fresh-peeled CWCs pulp was determined using the method of Liu et al. (2018), followed by grinding and pestling 10 g of fresh-peeled CWCs pulp. An LQ80T portable refractometer was used to measure the total soluble solid content.

The weight loss rate was measured using the method of Chen et al. (2022). The mass weighed on the 0th day was M_0_, and the weight weighed on the n day was M_n_. The weight loss rate of CWCs was calculated by $\frac{M_{0}-M_{n}}{M_{0}}\times 100\%$ .

Headspace gas composition in CWCs packages was measured using the method of Chen et al. (2022). Checkpoint 3 Portable Headspace Analyzer was used to measure O_2_ and CO_2_ contents.

### Total flavonoid, total phenolic, and soluble quinone content

The total flavonoid content was measured by referring to a modified method by Liu et al. (2018). Fresh-peeled CWCs pulp (5 g) was homogenized in 50 mL of 0.6 mL/mL ethanol, followed by centrifugation at 4°C for 10 min (10,000 ×g). Later, the supernatant (4 mL) was incubated with 0.3 mL of 50 g/L NaNO_2_ for 6 min and then 0.3 mL of 100 g/L Al (NO_3_)_3_ was added. After 6 min, 4 mL of 1 mol/L NaOH was added to the mixture with 0.8 mL/mL ethanol and incubated for 15 min. The absorbance was measured at 510 nm. The content of total flavonoids was indicated as mg kg^-1^.

The total phenolic content was quantified by using the method of Min et al. (2017). Fresh-peeled CWCs pulp (3 g) was mixed with 30 mL of 0.6 mL/mL ethanol, followed by homogenization for 6 min and centrifugation at 4 °C for 5 min (10,000 ×g). Then 0.125 mL of Folin-Ciocalteau and 1.25 mL of 7% Na_2_CO_3_ and 1 mL of distilled water were added, till the temperature reached 25°C for 90 min in the dark. The standard curve was determined with gallic acid solution. The absorbance was measured at 760 nm and the content of total phenolics was indicated as mg kg^-1^.

The soluble quinone content was determined by referring to the method of Homaida et al. (2017). Fresh-peeled CWCs pulp (3 g) was mixed with 20 mL of methanol, followed by homogenization for 1 min and centrifugation at 4°C for 10 min. The soluble quinone content was measured at 437 nm and indicated as A_437nm_ g^-1^.

### Enzyme activity assay (PAL, PPO, and POD)

The extraction and assay of PAL, PPO, and POD activity were conducted based on our previous paper (Min et al., 2019).

The PAL activity was measured using the Phenylalanine Ammonia Lyase Kit. Fresh-peeled CWCs pulp (0.3 g) was mixed with precooled reagent 1 (2.7 mL), followed by centrifugation at 4°C for 10 min (10,000 ×g). The subsequent procedures were conducted according to the manufacturer’s instructions. A unit of PAL activity was defined as a change in absorbance of 0.1 at 290 nm per fresh weight per min.

To determine the PPO activity, three grams of fresh-peeled CWCs pulp was mixed in 50 mL of phosphate buffer. Then they were centrifuged at 4 °C for 15 min (6,000 ×g). A unit of PPO activity was defined as a change in absorbance of 0.001 at 420 nm per min per fresh weight.

To determine the POD activity, five grams of fresh-peeled CWCs pulp was mixed with 5 mL of extraction buffer (1 mmol/L MPEG, 40 g/L PVPP, and 10 g/L Tritonx-100). Then they were ground and homogenized, followed by centrifugation at 4°C for 30 min (12,000 ×g). A unit of POD activity was defined as a change in absorbance of 0.01 at 470 nm per min per fresh weight.

### H_2_O_2_ content, O_2_·^-^ generation rate, OH·^-^ production rate, and MDA content

The hydrogen peroxide test kit was used to determine the H_2_O_2_ content. Two grams of fresh-peeled CWCs pulp and 18 mL of 8.5 g/L NaCl were diluted 10 times in an ice bath. Then they were centrifuged for 10 min at 4°C (10,000 ×g) and the supernatant was taken as the reaction solution. Finally, the absorbance value was measured at 405 nm and expressed as mmol g^-1^. O_2_·^-^ generation rate was measured by referring to the method (Zou et al., 2019). Two grams of fresh-peeled CWCs pulp was mixed with 5 mL of 50 mmol/L phosphate buffer and were ground in an ice bath. After that, they were centrifuged for 10 min at 4°C (12,000 ×g). Later, the supernatant was centrifuged at 12,000 ×g for 20 min. 1 mL of the supernatant was incubated with 10 mmol/L hydroxylamine hydrochloride and 5 mL of 50 mmol/L of phosphate buffer at 25°C for 20 min. They were then incubated at 25°C with 1 mL of 7 mmol/L α-naphthylamine and 1 mL of 17 mmol/L p-aminobenzene sulfonic acids. Finally, the absorbance value was measured at a wavelength of 530 nm and expressed as μmol g^-1^ min^−1^.

OH·^-^ production rate was measured using the hydroxyl radical kit (Nanjing Jiancheng Biological Engineering Co., Ltd., Nanjing, China). Five grams of fresh-peeled CWCs pulp was ground and homogenized with 20 mL of absolute ethanol, and they were centrifuged at for 10 min at 4°C (10,000 ×g). Then the supernatant was taken for experiments according to the kit instructions. Finally, the absorbance value was measured at a wavelength of 550 nm and expressed in nmol g^−1^ min^−1^.

The MDA content was determined by referring to the method of Kong et al. (2020). Three grams of fresh-peeled CWCs pulp was mixed with 15 mL of 100 g/L trichloroacetic acids, followed by centrifugation at 4°C for 20 min at 10,000 ×g. After that, 2 mL of the supernatant (corresponding to the blank control tube, added with 2 mL of 100 g/L trichloroacetic acid solution and 2 mL of 6.7 g/L thiobarbituric acids were mixed and placed in a water bath at 100 °C for 20 min. After cooling, the above centrifugation step was repeated. The absorbance was determined at 450, 532, and 600 nm, respectively. The MDA content was indicated as nmol g^-1^.

### CAT, SOD activity and DPPH free radical scavenging rate

The CAT activity was measured using a catalase test box kit (Nanjing Jiancheng Biological Engineering Co., Ltd., Nanjing, China). Two grams of fresh-peeled CWCs pulp and 18 mL of 8.5 g/L NaCl were diluted 10 times in an ice bath. They were then centrifuged for 10 min at 4°C (10,000 ×g) and the supernatant was taken as the reaction solution. Finally, the absorbance value was measured at 405 nm and indicated as U g^-1^.

The total superoxide dismutase test kit was used to measure the SOD activity. Five grams of fresh-peeled CWCs pulp was mixed with 20 mL of 0.15 mol/L phosphate buffer in the mortar. Next, they were ground under the condition of the ice bath and centrifuged at 10,000 ×g for 10 min. Then the supernatant was taken and diluted 7 times. The subsequent procedures were conducted according to the manufacturer’s instructions. Finally, the absorbance value was measured at a wavelength of 550 nm and indicated as U g^-1^.

The DPPH radical scavenging rate was measured using the method of Chen et al. (2022).Two grams of fresh-peeled CWCs pulp was homogenized in 25 mL of absolute ethanol in an ice bath, sonicated at 50°C for 30 min. After being centrifuged at 10,000 × g for 10 min at 4°C, and the supernatant were collected. The supernatant, DPPH alcohol solution and absolute ethanol solution were mixed in pairs. Then the reaction was performed at room temperature for 30 min in the dark, and the absorbance was measured at 517 nm.

### AsA, GSH content and APX, GR activity

The AsA content was measured using an ascorbate test kit (Nanjing Jiancheng Biological Engineering Co., Ltd., Nanjing, China). Five grams of fresh-peeled CWCs pulp and 20 mL of phosphate buffer, followed by homogenization for 3 min and centrifugation at 4 °C for 15 min (4,000 ×g). The supernatant was collected for testing. The subsequent procedures were conducted according to the manufacturer’s instructions. Finally, the absorbance value was measured at the wavelength of 536 nm and the results were indicated as μg g^-1^.

The GSH content was performed using a reduced glutathione test kit (Beijing Solarbio Science and Technology Co., Ltd., Beijing, China). The pulp of fresh-peeled CWCs were first washed twice with phosphate buffer, then 2 g of fresh-peeled CWCs pulp were homogenized in 5 mL of reagent 1 in an ice bath. They were then centrifuged for 10 min at 4°C (8,000 ×g). Then the supernatant was taken for experiments according to the kit instructions. Finally, the absorbance value was measured at 412 nm and the results were expressed as μg g^-1^.

The APX activity was measured using an ascorbate peroxidase test kit (Beijing Solarbio Science and Technology Co., Ltd., Beijing, China). Fresh-peeled CWCs pulp (1 g) was homogenized in 5 mL of reagent 1 in an ice bath and centrifuged at 13,000 ×g for 20 min at 4°C. Then the supernatant was taken for experiments according to the kit instructions. Finally, the absorbance value was measured at 290 nm and the results were indicated as U g^-1^.

The GR activity was measured using a glutathione reductase test kit (Beijing Solarbio Science and Technology Co., Ltd., Beijing, China). Fresh-peeled CWCs pulp (0.5 g) was homogenized in 5 mL of reagent 1 in an ice bath and centrifuged at 10,000 ×g for 10 min at 4°C. Then the supernatant was taken for experiments according to the kit instructions. Finally, the absorbance value was measured at 340 nm and the results were expressed as U g^-1^.

### Statistical analysis

The experiment was conducted three times and the findings were indicated as mean ± standard error. Before Duncan’s multiple range test, variance analysis (ANOVA) was used to compare means among groups. Analyses were performed by using the SPSS 19.0 software. Significance was expressed at *p*<0.05.

## Results

### The effect of EUG on quality indicators of fresh-peeled CWCs

Color difference is a key indicator for measuring the degree of the surface color change of fresh-cut vegetables and fruits (Valverde et al., 2005). Fresh-peeled CWCs treated with eugenol at a concentration of 0.75 g/L showed a slight discoloration (**Figure 1A**), while fresh-peeled CWCs treated with 1, 2 and 4 g/L eugenol had no obvious color change. Therefore, considering the effect and actual cost, 1 g/L eugenol was chosen for follow-up experiments. As shown in **Figure 1B**, eugenol treatment at a concentration of 1 g/L for 5 min delayed the discoloration of fresh-peeled CWCs in an effective manner.

**Figure 2:**
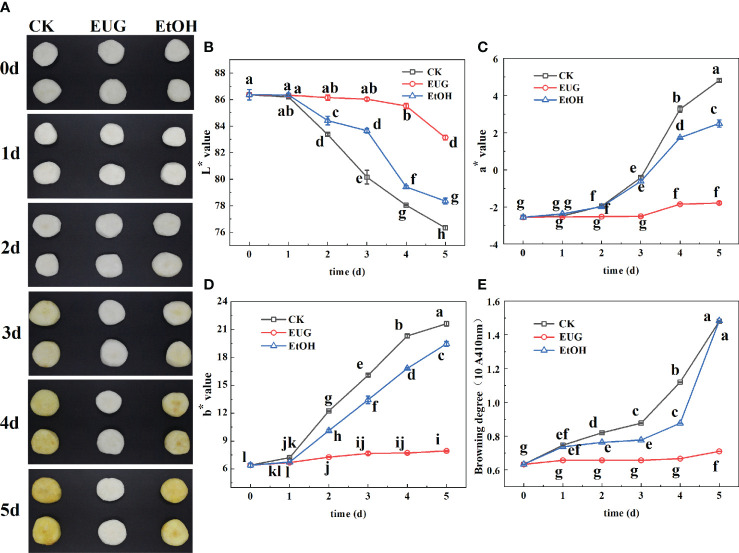
Effects of eugenol treatment on the visual quality **(A)**, color difference **(B–D)**, browning degree **(E)** of fresh-peeled CWC. Fresh-peeled CWCs were treated with tap water (CK), ethanol (EtOH) or eugenol (EUG). Error bars denote a standard error of the mean of triplicate assays. Different lowercase letters have shown significant differences (*P*<0.05).

The photos of the fresh-peeled CWCs soaked in distilled water, ethanol (5%) and eugenol solution (1 g/L) for 0-5 d (**Figure 2A**). L* values of all the eugenol, ethanol treatments and control groups showed a downward trend during storage (**Figure 2B**). The L* value of the control group (86.3-76.3) and the ethanol treatment group (86.3-78.4) were much lower than that of the eugenol treatment group (86.3-83.1) on 0-5 d, and there were significant differences from the second day. Throughout the storage, a* and b* values of fresh-peeled CWCs tended to increase, while a* and b* values of the eugenol treatment group were markedly lower than that of the control and ethanol treatment groups on 2-5 d (**Figure 2C, D**). After 5 d of storage, a* value in the control, ethanol treatment and eugenol treatment groups increased by 73.6%, 50.5% and 7.7%, respectively. And the b* value of the control and the ethanol treatment groups was 2.7 and 2.5-fold higher than that of the eugenol treatment group on 5 d.

The browning degree is one of the most important indicators to evaluate the quality of fruits and vegetables (Yang et al., 2021). The browning degree of fresh-peeled CWCs continued rising (**Figure 2E**), the control and ethanol-treated groups were significantly higher than those of the eugenol-treated group throughout the storage. The browning degree of the control and ethanol treatment group was 2.08-fold as high as that of the eugenol treatment on 5 d.

**Figure 3:**
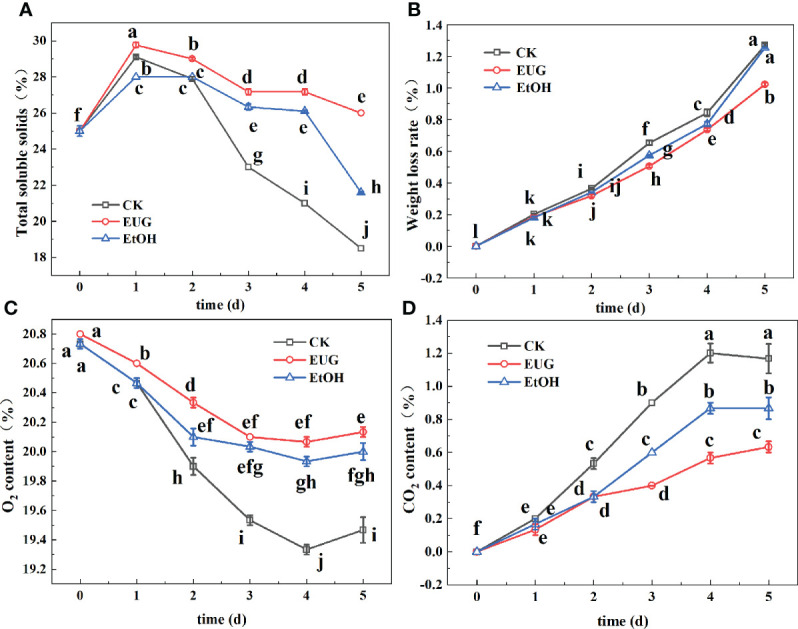
Effects of eugenol treatment on the total soluble solids **(A)**, weight loss rate **(B)**, O_2_ content **(C)** and CO_2_ content **(D)** of fresh-peeled CWC. Fresh-peeled CWCs were treated with tap water (CK), ethanol (EtOH) or eugenol (EUG). Error bars denote a standard error of the mean of triplicate assays. Different lowercase letters have shown significant differences (*P*<0.05).

Total soluble solids reflect the content of soluble sugar and other nutrients in fresh-peeled CWCs (Guerreiro et al., 2015). As shown in **Figure 3A**, the content of soluble solids in all groups increased rapidly on the 1st day and then decreased gradually on 2-5 d. The content of soluble solids in the eugenol treatment group was significantly higher than that of the control and ethanol treatment groups during the whole storage. By the end of storage, the total soluble solid content of fresh-peeled CWCs in the control and ethanol-treated groups decreased to 18.5 and 21.6%, while that of eugenol-treated fresh-peeled CWCs was 26%.

Fresh-peeled CWCs are mechanically damaged, resulting in the outflow of intracellular juices, and the weight continues to decrease during storage (Chen et al., 2017). As shown in **Figure 3B**, the weight loss rate of fresh-peeled CWCs in the three groups showed an upward trend during the whole storage period, the control and the ethanol treatment groups were significantly higher than the eugenol treatment group at the later storage period. The weight loss rates of the control and ethanol-treated groups on 5 d were 1.27% and 1.25% compared to 1.02% in the eugenol-treated group.

The partial pressures of O_2_ and CO_2_ reflect the respiration intensity of fresh-peeled CWCs (Chen et al., 2022). During the whole storage, the O_2_ content first decreased and then increased slowly (**Figure 3C**). From the first day, the eugenol-treated group was significantly higher than the control and ethanol-treated groups. The CO_2_ content showed a trend of first increase and then a slight decrease, and the eugenol treatment group was significantly lower than the control and ethanol treatment groups in the late storage period (**Figure 3D**).

### The effect of EUG on total flavonoids, total phenolics, and soluble quinones of fresh-peeled CWCs

**Figure 4:**
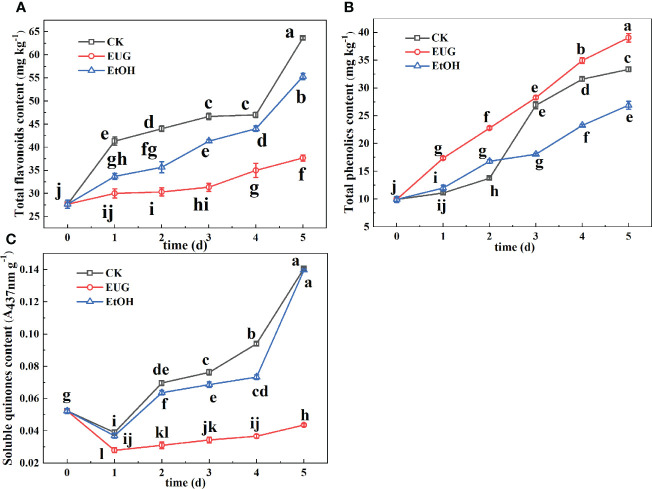
Effects of eugenol treatment on the total flavonoids **(A)**, total phenolics **(B)**, and soluble quinones **(C)** of fresh-peeled CWC. Fresh-peeled CWCs were treated with tap water (CK), ethanol (EtOH) or eugenol (EUG). Error bars denote a standard error of the mean of triplicate assays. Different lowercase letters have shown significant differences (*P*<0.05).

The yellowing of fresh-peeled CWCs may be due to the presence of total flavonoids (Pan et al., 2015). It can be seen from **Figure 4A** that the total flavonoid content of the control and ethanol-treated groups was significantly higher than that of the eugenol-treated group during the whole storage. Compared with eugenol-treated fresh-peeled CWCs, the total flavonoid content of the control and ethanol-treated groups increased rapidly during the later storage period. On the last day of storage, the total flavonoid content of the control and ethanol-treated groups rose to 63.7 and 55.3 mg kg^-1^, while that of the eugenol treatment group was 37.7 mg kg^-1^.

Phenolics are not key substrates of enzymatic browning in vegetables and fruits, but also have antioxidant effects (Teng et al., 2020). The total phenolic content of fresh-peeled CWCs showed an increasing trend (**Figure 4B**). The control and ethanol-treated groups were significantly lower than those of the eugenol-treated group in the early storage period. However, in the later storage period, the control group was lower than the eugenol-treated group but higher than the ethanol-treated group.

Quinones are the oxidation products of phenols during the enzymatic browning reaction (Saltveit, 2000). As shown in **Figure 4C**, the content of soluble quinone in the three groups showed a trend of first decreasing and then increasing, and the control and ethanol treatment group were significantly higher than eugenol treatment during the whole storage. For example, the content of soluble quinones in the control and ethanol-treated groups was 3.5-fold higher than that in the eugenol-treated group on 5 d.

### The effect of EUG on the enzymatic activity of fresh-peeled CWCs

**Figure 5:**
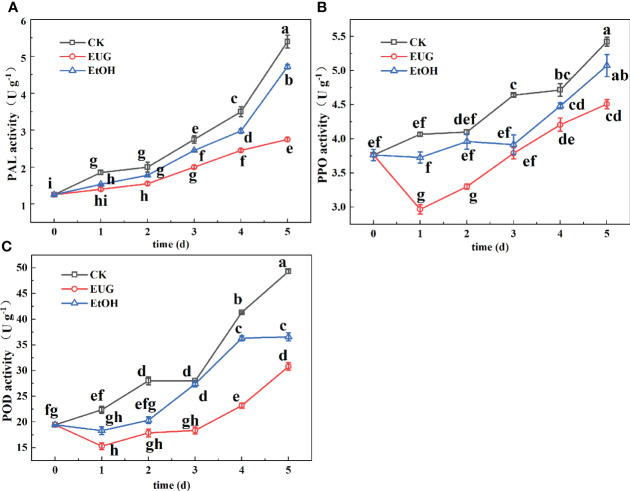
Effects of eugenol treatment on the activities of PAL **(A)**, PPO **(B)**, and POD **(C)** of fresh-peeled CWC. Fresh-peeled CWCs were treated with tap water (CK), ethanol (EtOH) or eugenol (EUG). Error bars denote a standard error of the mean of triplicate assays. Different lowercase letters have shown significant differences (*P*<0.05).

PAL is an important enzyme in the phenylpropane pathway for producing secondary metabolites such as flavonoids (Chen et al., 2017). It can be found from **Figure 5A** that the PAL activity of fresh-peeled CWCs showed an upward trend during the entire storage, and the PAL activity of the control and ethanol-treated groups was markedly higher than that of the eugenol-treated group. On the last day of storage, the PAL activity of the control and ethanol-treated groups rose to 5.4 and 4.7 U g^-1^, while the eugenol-treated group was only 2.75 U g^-1^.

PPO is one of the main causes of the enzymatic browning of vegetables and fruits (Teng et al., 2020). During the whole storage, the PPO activity of fresh-peeled CWCs in both the control and ethanol-treated groups was higher than that in the eugenol-treated group. Moreover, the control group was significantly higher than the eugenol-treated group (**Figure 5B**). The PPO activity in the control and ethanol treatment groups reached 5.4 and 5.1 U g^-1^ on 5 d, while that of the eugenol treatment group was 4.5 U g^-1^.

POD is an important enzyme in the process of enzymatic browning and melanin synthesis (Toivonen and Brummell, 2008). As shown in **Figure 5C**, the POD activity of the control and ethanol-treated groups was higher than that of the eugenol-treated group throughout the storage. There were significant differences between the eugenol-treated group and the control and ethanol-treated groups in the late storage. By the end of storage, the POD activity of the control and ethanol-treated groups increased by 60.6 and 46.9%, while the eugenol-treated group increased by 36.9%.

### The effect of EUG on H_2_O_2_ content, O_2_·^-^ generation rate, OH·^-^ production rate, and MDA content of fresh-peeled CWCs

**Figure 6:**
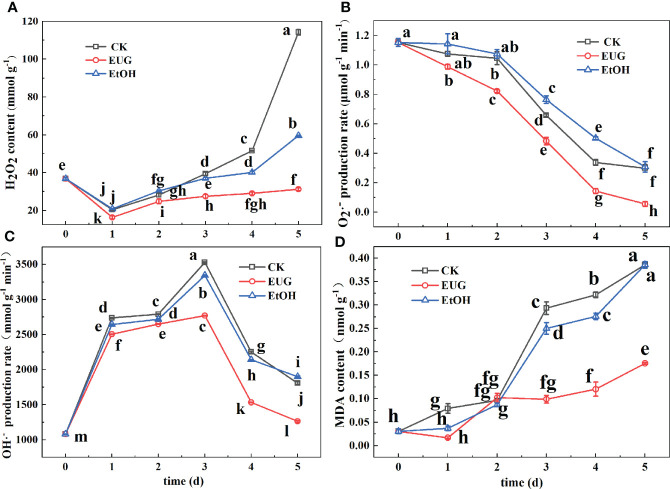
Effects of eugenol treatment on the H_2_O_2_ content **(A)**, O_2_·^-^ production rate **(B)**, OH·^-^ production rate **(C)** and MDA content **(D)** of fresh-peeled CWC. Fresh-peeled CWCs were treated with tap water (CK), ethanol (EtOH) or eugenol (EUG). Error bars denote a standard error of the mean of triplicate assays. Different lowercase letters have shown significant differences (*P*<0.05).

H_2_O_2_ can directly or indirectly oxidize biological macromolecules in the cell and destroy the cell membrane, accelerating cell senescence and disintegration (Asaeda et al., 2018). It can be seen from **Figure 6A** that throughout the whole storage, the H_2_O_2_ content of the control and ethanol treatment groups was markedly higher than that of the eugenol treatment group. And the H_2_O_2_ content of the control and the ethanol-treated group was 3.7 and 1.9-fold as high as that of the eugenol-treated group on 5 d, respectively. O_2_·^-^ is one of the most important reactive oxygen species (Kong et al., 2020). During the whole storage period, the O_2_·^-^ production rate of fresh-peeled CWCs showed a downward trend (**Figure 6B**). The O_2_·^-^ generation rate of the control and ethanol treatment groups was markedly higher than that of the eugenol treatment group on 2-5 d. During the whole storage, the O_2_·^-^ production rate in the control and ethanol-treated groups decreased from 1.15 to 0.29 and 0.30 μmol g^-1^ min^-1^, while that of the eugenol-treated group decreased from 1.15 to 0.05 μmol g^-1^ min^-1^.

OH·^-^ is one of the most oxidative free radicals in ROS, which can react with almost all cellular components and cause great damage to plants (Dou et al., 2021). It can be seen from **Figure 6C** that the OH·^-^ generation rate showed a trend of increasing first and then decreasing. And the eugenol-treated group was significantly lower than that of the control and ethanol-treated groups. In the last two days of storage, the OH·^-^ generation rate of the control and ethanol treatment groups was about 1.4-fold that of the eugenol treatment group.

MDA is one of the membrane lipid peroxidation products of the cell membrane (Kong et al., 2020). The MDA content of the control and ethanol-treated groups steadily rose during the storage (**Figure 6D**), but that of the eugenol-treated group first decreased and then increased. The control and ethanol-treated groups were significantly higher than the eugenol-treated groups on 3-5 d. On 5 d, the MDA content of the control and ethanol-treated groups was 2.17-fold that of the eugenol-treated group.

### The effect of EUG on CAT, SOD activity and DPPH free radical scavenging rate of fresh-peeled CWCs

**Figure 7:**
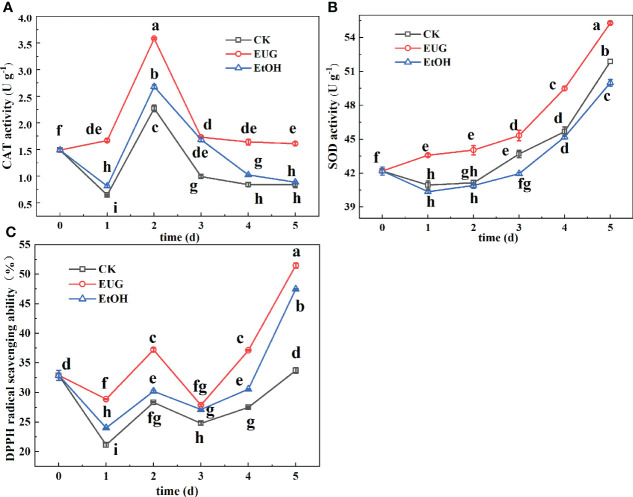
Effects of eugenol treatment on the activities of CAT **(A)**, SOD **(B)** and DPPH radical scavenging ability **(C)** of fresh-peeled CWC. Fresh-peeled CWCs were treated with tap water (CK), ethanol (EtOH) or eugenol (EUG). Error bars denote a standard error of the mean of triplicate assays. Different lowercase letters have shown significant differences (*P*<0.05).

CAT is present in peroxides, and its main function is to specifically catalyze the decomposition of H_2_O_2_ to generate H_2_O and O_2_ (Li et al., 2021). The CAT activity of fresh-peeled CWCs witnessed a fluctuation during the storage (**Figure 7A**). However, the CAT activity of the eugenol treatment group was significantly higher than that of the control and ethanol treatment groups throughout the storage. On 5 d, the eugenol-treated group was 1.9 and 1.8-fold more than the control and ethanol-treated groups.

The catalytic effect of SOD is to inhibit the damage of O_2_·^-^ to the plant body and establish the first line of defense to the antioxidant system (Peskin and Winterbourn, 2000). The SOD activity of the control and ethanol-treated fresh-peeled CWCs decreased first and then increased during the storage (**Figure 7B**), while the eugenol-treated group showed significantly higher SOD activity compared with that of the control and ethanol-treated groups. By the end of storage, the SOD activity in the eugenol treatment group reached 55.28 U g^-1^, while the control and ethanol-treated groups were 51.87 and 49.99 U g^-1^. Eugenol treatment increased SOD activity, inhibited the production of harmful substances and enhanced antioxidant capacity in fresh-peeled CWCs.

DPPH free radical scavenging rate is one of the indicators to evaluate the antioxidant capacity of plants (Chen et al., 2022). The DPPH free radical scavenging rate of fresh-peeled CWCs showed a fluctuating upward trend (**Figure 7C**). During the whole storage, the eugenol treatment group was consistently higher than the control and ethanol treatment groups, and there was a significant difference between the eugenol-treated group and the control group. On 5 d, the DPPH free radical scavenging rate in the eugenol-treated group was 51.5%, compared with 33.73% in the control group.

### The effect of EUG on AsA, GSH content and APX, GR activity of resh-peeled CWCs

**Figure 8:**
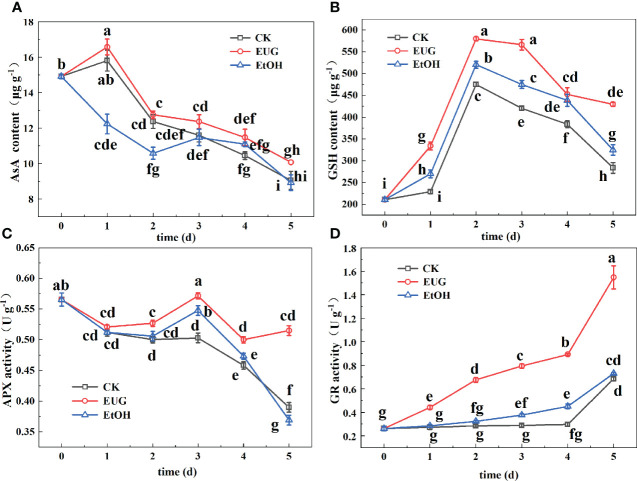
Effects of eugenol treatment on the AsA content **(A)**, GSH content **(B)**, APX activity **(C)** and GR activity **(D)** of fresh-peeled CWC. Fresh-peeled CWCs were treated with tap water (CK), ethanol (EtOH) or eugenol (EUG). Error bars denote a standard error of the mean of triplicate assays. Different lowercase letters have shown significant differences (*P*<0.05).

The AsA-GSH cycle is mainly composed of the interaction of AsA, GSH, APX and GR, and plays an important role in scavenging ROS. AsA is a vitamin substance widely distributed in plant tissues. It participates in the redox effect in the electron transport system in plants and is an important reducing agent in non-enzymatic antioxidant system of plants (Xu et al., 2022). The AsA content in fresh-peeled CWCs generally showed a downward trend (**Figure 8A**). The eugenol-treated group was higher than the control and ethanol-treated groups, and there was a significant difference between the eugenol-treated group and the control group from the third day to the end of storage. On the last day, the AsA content decreased by 39.3% and 40.2% in the control and ethanol-treated groups, respectively, while the eugenol-treated group decreased by 32.5%.

As a common non-enzymatic antioxidant, GSH not only directly scavenges a range of ROS, but also participates in many other functions that keep cells in a favorable state (Wang et al., 2021). It can be seen from **Figure 8B** that the GSH content in fresh-peeled CWCs first increased and then decreased. During the entire storage period, the GSH content of the eugenol-treated group was higher than that of the other two groups, and there were significant differences between the control and eugenol-treated groups. On 5 d, the eugenol treatment group was 1.5 and 1.3-fold as high as the control and ethanol treatment groups.

APX and GR are antioxidant enzymes that work together with antioxidant substances to remove ROS to protect the integrity of the cell membrane (Xu et al., 2022). The APX activity of eugenol treatment group was higher than the other two groups. During the whole storage, and there was a significant difference in the later storage (**Figure 8C**). On 5 d, the eugenol-treated groups were 1.3 and 1.4-fold as high as the control and ethanol-treated groups, respectively.

The GR activity in the three groups showed an upward trend, and the eugenol-treated group was significantly higher than the control and ethanol-treated groups during the entire storage (**Figure 8D**). On the last day of storage, the eugenol-treated group rose to 1.55 U g^-1^, while the control and ethanol-treated groups were 0.68 and 0.73 U g^-1^.

### Correlation analysis

**Figure 9:**
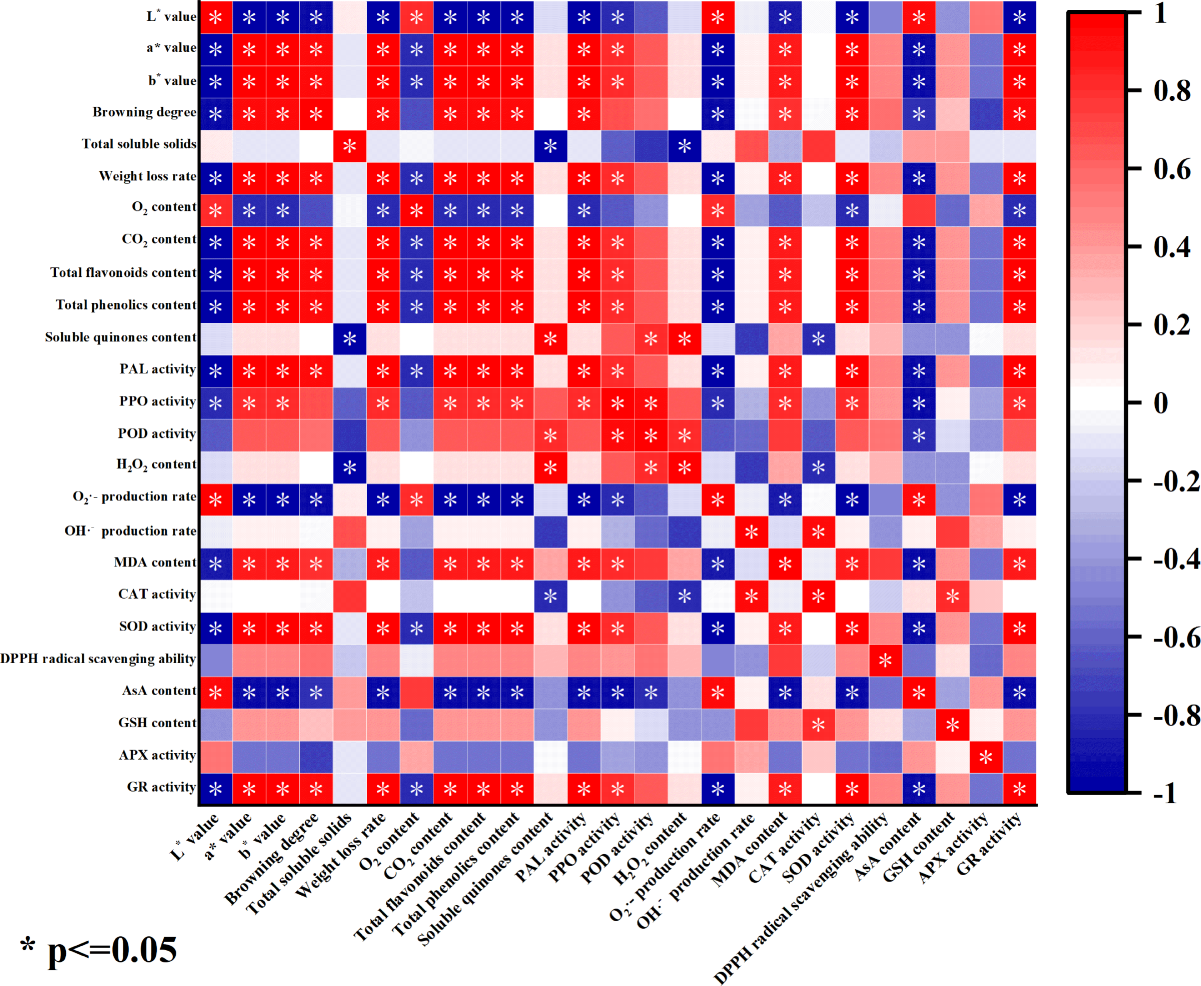
Correlation heat map of each index of fresh-peeled CWCs treated with eugenol. (The color depth indicates the strength of the correlation, the redder the color, the stronger the positive correlation, and the bluer the color, the stronger the negative correlation).

The color change of fresh-peeled CWCs could directly reflect its storage quality (Valverde et al., 2005). As can be seen from **Figure 9**, the color difference L* value of fresh-peeled CWCs treated with eugenol was significantly positively correlated with O_2_ content, O_2_·^-^ generation rate and AsA content. This suggests that eugenol treatment may delay the quality deterioration of fresh-peeled CWCs by inhibiting the rate of O_2_·^-^ generation, reducing respiration intensity and loss of ascorbic acid. The color difference L* value was significantly negatively correlated with a*, b* value, browning degree, weight loss rate, the contents of CO_2_, total flavonoids, total phenolics, and MDA, antioxidant enzyme activities (PAL, PPO, SOD, and GR). This indicated that the eugenol treatment may reduce the degree of browning and weight loss rate, and improving the antioxidant capacity, thereby inhibiting the discoloration of fresh-peeled CWCs and prolonging its shelf life.

## Discussion

Due to the mechanical damage during the fresh-cut processing, fresh-peeled CWCs suffer quality deterioration, such as discoloration and nutrient loss (Song et al., 2019). The appearance of fresh-peeled CWCs gradually deteriorated during storage, which is consistent with the increase in a* and b* values and the decrease in the L* value. These results accorded with the changes in browning degree in fresh-peeled CWCs. Thus, eugenol treatment delayed the discoloration of fresh-peeled CWCs, which is similar to the results of eugenol treatment in eggplant fruit (Huang et al., 2019), fresh-cut lettuce (Chen et al., 2017), citrus fruit (Yang et al., 2021) and cucumber (Li et al., 2021). Meanwhile, the total soluble solids reflect the content of soluble sugar and other nutrients in fresh-peeled CWCs (Song et al., 2019). It is found that eugenol treatment effectively maintained the total soluble solids level of fresh-peeled CWCs, which is consistent with the results of eugenol treatment in eggplant fruit (Huang et al., 2019) and citrus fruit (Yang et al., 2021). It may be due to that eugenol treatment accelerates the converting of organic acids and starches to soluble sugars (Yang et al., 2021). However, fresh-peeled CWCs are susceptible to mechanical damage, and the respiration and transpiration are enhanced, so the water loss will increase and the weight loss rate will increase. Eugenol treatment not only delayed the loss of the total soluble solids content in fresh-peeled CWCs, but also reduced the loss of juice of its fruit, which reduced the weight loss rate of fresh-peeled CWCs treated with eugenol. Eugenol treatment reduced the weight loss rate of fresh-peeled CWCs, which was similar to the findings of eugenol-treated fresh-cut lettuce (Chen et al., 2017) and cucumber fruit (Li et al., 2021). The headspace gas composition could reflect the respiration intensity of fresh-peeled CWCs. The O_2_ content was higher and the CO_2_ content was lower after eugenol treatment. Therefore, eugenol treatment inhibited the respiration intensity of fresh-peeled CWCs and delayed the quality deterioration. And this is consistent with the results of eugenol-treated grape fruit (Valverde et al., 2005). In addition, many researchers have found that eugenol treatment can inhibit the growth of microorganisms, indicating that eugenol treatment has antiseptic and antibacterial effects (Zheng et al., 2013). Therefore, eugenol treatment could inhibit the respiration intensity of fresh-cut fruits and vegetables to maintain the quality of fresh-cut fruits and vegetables.

Phenolic metabolism is another important cause of discoloration (Pen and Jiang, 2003). After mechanical damage, the phenolic metabolism of fresh-cut fruits and vegetables will be activated (Pen and Jiang, 2003). PAL is an important enzyme in the metabolic pathway of phenylpropane for connecting phenylpropane metabolism with primary metabolism and catalyzing phenylalanine to generate cinnamic acids, followed by the production of flavonoids and other secondary metabolites (Min et al., 2017). Likewise, according to the study treating fresh-cut water chestnuts with exogenous ascorbic acid and ferulic acid, PAL is the key enzyme to promoting discoloration (Song et al., 2019). Throughout the storage, the PAL activity of fresh-peeled CWCs treated with eugenol was markedly lower than that of the control group (*P*<0.05). The total phenolics are key substrates that cause discoloration of fresh-cut vegetables and fruits, and also play antioxidant effects (Dou et al., 2021). In our study, eugenol treatment increased the total phenolic content of fresh-peeled CWCs, which is consistent with the results of eugenol treatment in eggplants (Huang et al., 2019) and tomatoes (Mirdehghan and Valero, 2017), and the active package containing eugenol for organic ready-to-eat iceberg lettuce (Wieczyńska and Cavoski, 2018). Moreover, in the study of fresh-peeled CWCs treated with 15 g/L eugenol emulsion, the eugenol emulsion treatment group exhibited higher content of p-hydroxybenzoic acid and chlorogenic acid (Teng et al., 2020). It may be that the increase in the content of these monophenols such as p-hydroxybenzoic acid and chlorogenic acid led to the increase in the total phenolic content of fresh-peeled CWCs. Under normal circumstances, the browning of vegetables and fruits is caused by the oxidation of phenolic substances into quinones, catalyzed by POD and PPO in the presence of oxygen (Huang et al., 2019). Quinones polymerize spontaneously and react with the side-chain groups of protein amino acid residues to produce black or brown substances (Toivonen and Brummell, 2008). The POD and PPO activity of fresh-peeled CWCs treated with eugenol were markedly lower than those of the control group during the whole storage (*P*<0.05), which is consistent with the results of fresh-cut lettuce treated with eugenol (Chen et al., 2017). Similar to the suppressed PPO and POD activity, fresh-peeled CWCs treated with eugenol showed lower total content of soluble quinones. However, some researchers believe that the cause of fresh-peeled CWCs discoloration is yellowing instead of enzymatic browning and that flavonoids such as naringenin and eriodictyol are the major substances of yellowing (Pan et al., 2015; Song et al., 2019). Throughout the storage, the total flavonoid content of fresh-peeled CWCs treated with eugenol was markedly lower than that of the control group (*P*<0.05). This suggests that eugenol treatment may delay the discoloration of fresh-peeled CWCs by inhibiting the production of total flavonoids. Therefore, eugenol treatment not only inhibited the production of flavonoids and quinones to a certain extent, but also reduced the production of PAL, PPO, POD. Moreover, eugenol treatment promoted the production of some phenolic substances, improved the antioxidant capacity of fresh-cut fruits and vegetables, and inhibited their discoloration.

The imbalance of ROS metabolism is an important reason for causing discoloration of vegetables and fruits during fresh-cutting (Tripathy and Oelmüller, 2012; Li et al., 2022). ROS mainly consists of H_2_O_2_, superoxide radicals (O_2_·^-^), hydroxyl radicals (OH·^-^) and lipid peroxides (MDA). H_2_O_2_ can directly or indirectly oxidize biological macromolecules in cells, such as nucleic acids and proteins, thereby accelerating cell senescence and disintegration (Asaeda et al., 2018). The increase in O_2_·^-^ generation will cause membrane lipid peroxidation, thereby accelerating the senescence of plant tissues (Kong et al., 2020). OH·^-^ is one of the most oxidative free radicals that can react with all cells and cause damage to the plant body (Dou et al., 2021). As one of the membrane lipid peroxidation products of cell membranes, MDA can damage the cell membrane of plant tissues and accelerate discoloration (Kong et al., 2020). During the whole storage process, the contents of H_2_O_2_ and MDA and the production rate of O_2_·^-^ and OH·^-^ in fresh-peeled CWCs treated with eugenol were markedly lower than those in the control group (*P*<0.05). This finding is consistent with research results of eugenol treated eggplant fruit (Huang et al., 2019) and cucumbers treated by double layer membrane loading eugenol (Li et al., 2021). So, the eugenol treatment inhibited the accumulation of these harmful substances. There is a complete ROS scavenging system in vegetables and fruits. It mainly including antioxidant enzymes and antioxidants (Li et al., 2021), which can not only exert their own antioxidant effects, but also coordinate with each other to scavenge ROS. A main function of CAT is to catalyze the decomposition of H_2_O_2_ to generate H_2_O and O_2_ (Li et al., 2021). SOD is the first line of defense to inhibit the damage of O_2_·^-^ on plant bodies and establish an antioxidant system (Yang et al., 2021). During the whole storage process, the fresh-peeled CWCs treated with eugenol showed higher CAT and SOD activities than the control group (*P*<0.05). Similarly, SOD and CAT activity were markedly enhanced in citrus fruits treated by eugenol nano-emulsion (Yang et al., 2021) and eugenol emulsion-treated fresh-cut water chestnuts (Zhu et al., 2022). DPPH free radical scavenging rate is closely related to plant antioxidant capacity (Chen et al., 2022). It can be seen from the experimental results that the DPPH free radical scavenging rate in the eugenol treatment group was significantly higher than that in the control group (*P*<0.05). This result was similar to that of strawberry treated with eugenol (Wang et al., 2007), which also enhanced the DPPH free radical scavenging rate. Therefore, eugenol treatment improved the antioxidant capacity of fresh-peeled CWCs. The AsA-GSH cycle is mainly composed of the interaction of AsA, APX, GSH and GR, and plays an important role in scavenging ROS. During the whole storage process, the AsA and GSH contents and APX and GR activities of fresh-peeled CWCs treated with eugenol were higher than those of the control and ethanol-treated groups. This is similar to the results of treating fresh-cut CWCs with hydrogen sulfide (Dou et al., 2021), fresh-cut pears treated with high carbon dioxide (Wang et al., 2021), and fresh-cut pitaya fruit pretreated with hot air (Li et al., 2022). Therefore, eugenol treatment results in an increase the contents of these antioxidants and the activities of antioxidant enzymes in fresh-peeled CWCs. Moreover, eugenol treatment could inhibit the production of ROS metabolism, thereby inhibiting the spoilage and aging of fresh-cut fruits and vegetables, and reducing the accumulation of harmful substances. Meanwhile, eugenol treatment could promote the generation of antioxidant enzymes and antioxidants in the ROS scavenging system to maintain the quality of fresh-cut fruits and vegetables, and play a very important role in extending their shelf life.

## Conclusions

This study indicated that eugenol treatment could effectively delay the surface discoloration, reduce the degree of browning, weight loss rate and the loss of soluble solids and increase the O_2_ content, reduce the CO_2_ content in fresh-peeled CWCs. Moreover, eugenol treatment markedly decreased the content of total flavonoids and soluble quinones and inhibited the activity of POD, PPO, and PAL of fresh-peeled CWCs. In addition, eugenol treatment increased the total phenolic content and DPPH radical scavenging rate, and improved the antioxidant ability of fresh-peeled CWCs. In particular, the ROS production (H_2_O_2_, O_2_·^-^ and OH·^-^) and MDA level were suppressed by eugenol treatment, while promoting the production of antioxidants (AsA and GSH), and enhanced the activities of antioxidant enzymes (CAT, SOD, APX and GR). In summary, the fresh-peeled CWCs after eugenol treatment can effectively maintain the storage quality by inhibiting the phenolic metabolism, improving the antioxidant capacity and scavenging the ROS. Thus, eugenol treatment has broad potential for the preservation of fresh-cut vegetables and fruits in future.

## Data availability statement

The original contributions presented in the study are included in the article/Supplementary Material. Further inquiries can be directed to the corresponding author.

## Author contributions

ZC and TM designed the study; ZC, YL and ZM carried out the experiments; ZC, TM, YX and YY made the data analysis; TM, ZC and WH drafted the manuscript; LW, TM, YA and HW reviewed the final manuscript. All authors contributed to the article and approved the submitted version.

## Funding

Supported by National Natural Science Foundation of China (no. 32001764).

## Conflict of interest

The authors declare that the research was conducted in the absence of any commercial or financial relationships that could be construed as a potential conflict of interest.

## Publisher’s note

All claims expressed in this article are solely those of the authors and do not necessarily represent those of their affiliated organizations, or those of the publisher, the editors and the reviewers. Any product that may be evaluated in this article, or claim that may be made by its manufacturer, is not guaranteed or endorsed by the publisher.
